# Human landing catches provide a useful measure of protective efficacy for the evaluation of volatile pyrethroid spatial repellents

**DOI:** 10.1186/s13071-023-05685-5

**Published:** 2023-03-07

**Authors:** Mgeni Mohamed Tambwe, Ummi Abdul Kibondo, Olukayode Ganiu Odufuwa, Jason Moore, Ahmed Mpelepele, Rajabu Mashauri, Adam Saddler, Sarah Jane Moore

**Affiliations:** 1 Vector Control Product Testing Unit, Ifakara Health Institute, P.O. Box 74, Bagamoyo, Tanzania; 2 Department of Epidemiology and Public Health, Swiss Tropical and Public Health Institute, Kreuzstrasse 2, 4123 Allschwill, Basel, Switzerland; 3 University of Basel, Petersplatz 1, 4001 Basel, Switzerland; 4 Telethon Kids Institute, Perth, Australia; 5 London School of Hygiene and Tropical Medicine, Keppel Street, London, WC1E 7HT UK; 6 Nelson Mandela African Institution of Science and Technology (NM-AIST), P.O. Box 447, Tengeru, Tanzania

**Keywords:** Ambient chamber, Semi-field system, Transfluthrin, Volatile pyrethroid, Passive emanator, *Aedes aegypti*, *Anopheles gambiae* sensu stricto, *Anopheles funestus* sensu stricto, Human landing catch, Bioassay

## Abstract

**Background:**

The human landing catch (HLC) method, in which human volunteers collect mosquitoes that land on them before they can bite, is used to quantify human exposure to mosquito vectors of disease. Comparing HLCs in the presence and absence of interventions such as repellents is often used to measure protective efficacy (PE). Some repellents have multiple actions, including feeding inhibition, whereby mosquitoes may be unable to bite even if they land on a host. A comparison was made between the PE of the volatile pyrethroid spatial repellent (VPSR) transfluthrin determined using a landing method (HLC) and a biting method (allowing the mosquitoes that landed to blood-feed) to evaluate whether HLC is a suitable method for the estimation of the personal PE of a VPSR.

**Methods:**

A fully balanced, two-arm crossover design study was conducted using a 6 × 6 × 2-m netted cage within a semi-field system. Hessian strips (4 m × 0.1 m) treated with a 5-, 10-, 15-, or 20-g dose of transfluthrin were evaluated against a paired negative control for three strains of laboratory-reared *Anopheles* and *Aedes aegypti* mosquitoes. Six replicates were performed per dose using either the landing or the biting method. The number of recaptured mosquitoes was analysed by negative binomial regression, and the PEs calculated using the two methods were compared by Bland–Altman plots.

**Results:**

For *Anopheles*, fewer mosquitoes blood-fed in the biting arm than landed in the landing arm (incidence rate ratio = 0.87, 95% confidence interval 0.81–0.93, *P* < 0.001). For *Ae. aegypti*, biting was overestimated by around 37% with the landing method (incidence rate ratio = 0.63, 95% confidence interval 0.57–0.70, *P* = 0.001). However, the PEs calculated for each method were in close agreement when tested by the Bland Altman plot.

**Conclusions:**

The HLC method led to underestimation of mosquito feeding inhibition as a mode of action of transfluthrin, and there were species- and dose-dependent differences in the relationship between landing and biting. However, the estimated PEs were similar between the two methods. The results of this study indicate that HLC can be used as a proxy for personal PE for the evaluation of a VPSR, especially when the difficulties associated with enumerating blood-fed mosquitoes in a field setting are taken into consideration.

**Graphical Abstract:**

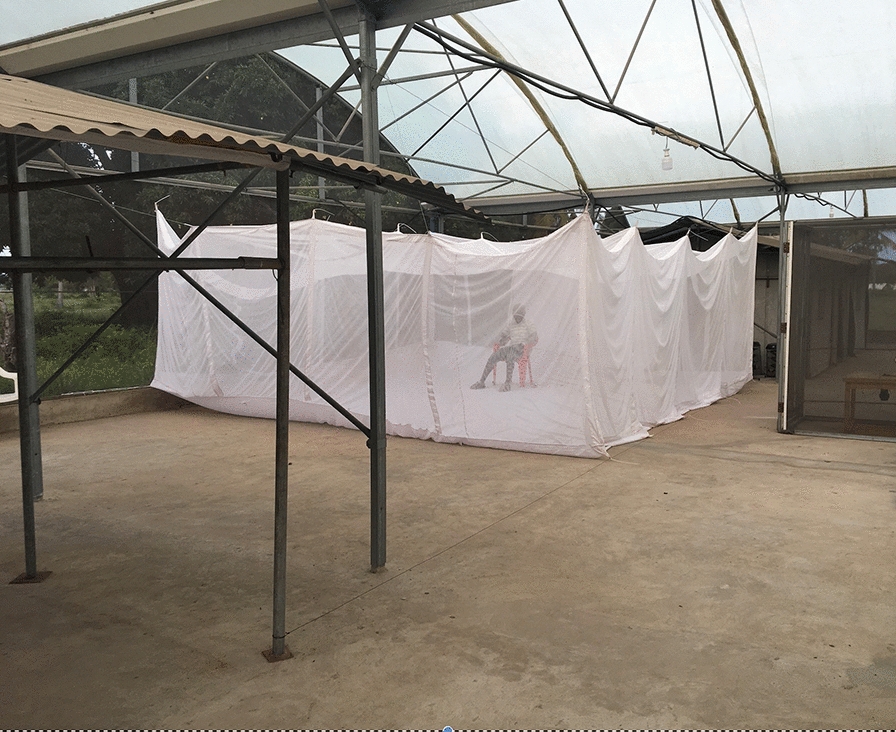

## Background

Appropriate and effective vector control tools are integral components of mosquito-borne disease control programs worldwide [1]. However, incomplete coverage and poor compliance of vector control interventions remain major challenges in the control of malaria [2] and arbovirus vectors [3]. In addition, some malaria and arbovirus vector species are not completely controlled by current insecticidal tools because they are either behaviourally resistant (they avoid contact with insecticides through outdoor biting or resting, or biting during the day) or physiologically resistant (they can survive contact with an insecticide) [4, 5]. The most efficient vectors of malaria and arboviruses are highly adapted to humans (synanthropic) and are therefore most commonly encountered around human dwellings, either indoors [6] or in the peridomestic space [7]. The former has been a major target location for malaria control for the last three decades through the use of insecticide-treated nets and indoor residual spraying [8], but targeting indoor spaces only is insufficient for the elimination of malaria in many sub-Saharan Africa regions [9]. Therefore, also targeting the peridomestic space with vector control interventions for outdoor biting mosquitoes is more effective as a strategy because many people in these regions spend an extensive amount of time outdoors for domestic activities, where they are unprotected against biting mosquitoes, which may explain residual malaria transmission in these areas [10]. Ideally, novel control interventions deployed in the peridomestic space should prevent bites and kill mosquitoes to provide both personal and community protection for users and non-users of the space [11]. The efficacy of volatile pyrethroid spatial repellents (VPSRs) as a means of protection against mosquitoes in the peridomestic space remains an unanswered research question, and robust methods for their evaluation in this setting are needed.

The semi-field system (SFS) was developed to evaluate the efficacy of vector control tools in a controlled disease-free environment [12]. This bioassay provides a convenient alternative method for the evaluation of vector control tools, and avoids some of the difficulties associated with field trials, such as variation in mosquito density, and the size and layout of houses [13]. SFS has been used to demonstrate the efficacy of VPSRs [14, 15] through the measurement of multiple outcomes, including blood-feeding inhibition, delayed resumption of feeding (disarming), delayed mortality, deterrence and fecundity reduction [16]. However, to maximize the precision of measurement of some endpoints, such as blood-feeding inhibition and delayed mortality, it is necessary to recapture all of the mosquitoes that may be encountered during an intervention. The Ifakara large ambient chamber test (I-LACT) is a large cage fitted inside an SFS with an area that approximates that of a typical peridomestic space, and was designed to improve the recapture of released mosquitoes. Outdoor vector control tools with multiple actions that impact mosquito feeding, and induce sublethal incapacitation or delayed mortality, may be more accurately assessed by using the I-LACT.

The human landing catch (HLC) method is a procedure whereby human volunteers catch mosquitoes that land on them before they bite, by using a mouth aspirator [17]. This procedure is usually used to estimate the protective efficacy (PE) of bite prevention interventions, such as repellents [18–20]. Repellents, particularly volatile pyrethroids, exhibit various modes of action, including interference with mosquito olfaction so that not all mosquitoes that land on a host are able to bite. Thus, HLC may underestimate the full PE of a bite-prevention intervention that modulates mosquito host perception [21] or blood-feeding behaviour [22]. Therefore, a comparison of PE of the VPSR transfluthrin was conducted in an I-LACT using either HLC (hereafter ‘landing’) or by allowing mosquitoes to freely interact with a volunteer and blood-feed on them (hereafter ‘biting’).

## Methods

### Description of the I-LACT

**Fig. 1:**
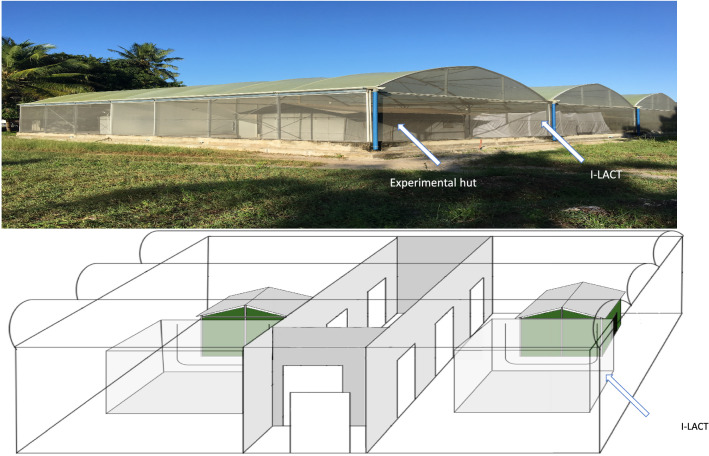
Photograph and diagram showing the semi-field system with an Ifakara large ambient chamber test (I-LACT; 6 × 6 × 2 m) in each compartment

The I-LACT in which the experiment was conducted is a polyester net cage measuring 6 × 6 × 2 m fixed inside an SFS located at the Ifakara Health Institute, Bagamoyo-Kingani, Tanzania (Fig. 1). The I-LACT dimensions represent the approximate size of the peridomestic space around rural Tanzanian homes, where most domestic activity occurs [23]. This bioassay was designed to ensure the maximum recovery of released mosquitoes for the evaluation of vector control tools. Preliminary experiments have shown that the recapture rate for the I-LACT is approximately 90%, whereas that of the standard SFS compartment is approximately 60%. The lower recapture rate in the SFS is due to its high roof and textured surfaces, which make it difficult to reach and see all released mosquitoes. The sides and roof of the I-LACT are made of polyester netting, to allow airflow, both floor and netting are white coloured to facilitate mosquito collection after exposure as mosquitoes can be easily seen against the white background. The compartment is sealed with a zip to prevent mosquito escape, and is kept free of mosquito predators through daily clearing of spiders and the use of sugar baits spiked with boric acid to minimize scavenging ants. The I-LACT enables controlled experiments with the simultaneous release of multiple laboratory mosquito strains to be carried out. In addition, as laboratory-reared mosquitoes are disease-free, conducting these experiments with blood-feeding endpoints is considered safe. For the experiment reported here, two I-LACTs were used, one for the treatments and one for the controls.

### Mosquitoes

Four strains of laboratory-reared mosquitoes were used in the experiments: the fully pyrethroid-susceptible *Anopheles gambiae* sensu stricto (s.s.) Ifakara strain; the pyrethroid-resistant (knock-down resistance; KDR) *Anopheles gambiae* s.s. Kisumu strain; the pyrethroid-resistant (metabolic resistance) *Anopheles funestus* FUMOZ strain; and the pyrethroid-susceptible *Aedes aegypti* Bagamoyo strain (Table 1). Colonies of these strains are maintained according to MR4 guidelines [24]. The larvae are fed on TetraMin fish flakes (Tetra, UK), and adults on 10% sugar ad libitum; females are membrane-fed cow’s blood for egg production. The colonies are maintained under approximately 12-h:12-h light:dark (natural light) at 27 ± 5 °C and 70 ± 30% relative humidity (RH).

**Table 1:** Results of the World Health Organization susceptibility test for the laboratory-reared mosquitoes used in this experiment

Mosquito species (strain)	24-h mortality					
	Permethrin (0.75%)	Deltamethrin (0.05%)	$\alpha$-cypermethrin (0.05%)	$\lambda$-cyhalothrin (0.05%)	Bendiocarb (0.1%)	Pirimiphos methyl (0.25%)
*Anopheles gambiae* (Ifakara)	94%	100%	100%	100%	100%	100%
*Anopheles gambiae* (Kisumu)^a^	88%	96%	72%	66%	94%	100%
*Anopheles funestus* (FUMOZ)	40%	38%	13%	100%	96%	100%
*Aedes aegypti* (Bagamoyo)	100%	100%	100%	100%	96%	100%

^a^Knock-down resistant (KDR)

Nulliparous 3–8 day-old mosquitoes were used for the experiments. Mosquitoes were selected by placing a hand near to their cage, and those that attempted to aggressively bite were aspirated into paper cups. When two mosquito strains of similar morphology were released simultaneously, red fluorescent pigment (Swada, Cheshire, UK) was used to mark the individuals of one of the strains so that the strains could be distinguished between. Mosquitoes were marked by dusting the mesh lid of the cup with a brush to create a cloud of pigment that was deposited onto the mosquitoes. After marking, the mosquitoes were aspirated into 10 × 10 × 10-cm release cages. The mosquitoes were transferred from the insectary to the SFS in a black cloth bag to prevent them from being damaged by the wind. *Aedes* mosquitoes were sugar starved for 12 h and *Anopheles* mosquitoes for 6 h prior to commencement of the experiments, to maximise their avidity without inducing excess mortality. Before each experiment, the mosquitoes were acclimatized for 45 min in the corridor of the SFS, which is separated from the experimental space by polyurethane sheeting to prevent the mosquitoes from coming into contact with the tested insecticides.

### World Health Organization susceptibility bioassays using transfluthrin-treated paper

Physiological susceptibility tests for transfluthrin were conducted for each mosquito strain before the start of semi-field experiments. The tests were performed using tube test bioassays following World Health Organization (WHO) guidelines [25]. As there is no recommended discriminating dose of transfluthrin for testing the susceptibility status of these mosquitoes, transfluthrin-impregnated papers at the doses proposed by Sukkanon et al. [26] were used. Five serial dilutions of emulsifiable concentrate (EC) were prepared by mixing with acetone and silicone oil in individual Falcon tubes. The concentrations of EC transfluthrin were 0.00125%, 0.0025%, 0.005%, 0.01%, 0.02%, 0.04%, 0.08% and 0.1% for *Anopheles*, and 0.003125%, 0.00625%, 0.125%, 0.025%, 0.05% and 0.1% for *Ae. aegypti*. Whatman grade 1 filter papers (12 × 15 cm; Whatman International, Banbury, UK) were prepared by impregnation with the concentrations of EC transfluthrin. For each filter paper**,** 2 ml of diluted EC transfluthrin was used. The impregnated papers were air-dried in the shade at ambient temperature, then wrapped in aluminium foil and refrigerated at 4 °C before use in the tests that were carried out on the same day. The papers were destroyed after the experiment.

One hundred and fifty non-blood-fed, 3–5-day-old mosquitoes were exposed to the transfluthrin-treated paper or to the control for 1 h. The mosquitoes were then provided with 10% sucrose solution and maintained at approximately 27 °C and 80% RH for the determination of 24 h mortality. Each dilution was tested four times.

The discriminating concentration (DC) for* Anopheles* (Table 4) was used to test the susceptibility status of *An. gambiae* (Kisumu strain; KDR) and *An. funestus* (FUMOZ strain). The same procedure was used as in the susceptibility test, and the same numbers of mosquitoes were exposed to the transfluthrin-treated paper as per the obtained DC.

### Preparation of the transfluthrin passive emanator

Hessian sacks (made from fibre of *Corchorus olitorius*) were purchased locally, washed using detergent powder (OMO) and water, and dried under direct sunlight. A concentration series of EC transfluthrin (Bayothrin EC; Bayer, Monheim am Rhein, Germany) was prepared. Eave-positioned targeted insecticide (EPTI) emanators comprising 4 m × 0.1 m strips of hessian treated with 5 g, 10 g, 15 g, or 20 g of transfluthrin [27] were used for the experiments with *Anopheles*. For *Aedes* mosquitoes, freestanding transfluthrin passive emanators (FTPEs) [28] comprising 5 m × 0.1 m hessian strips treated with the same four doses of transfluthrin were used. Negative controls were prepared in the same way with water.

### Study procedure

#### Experimental design

**Fig. 2:**
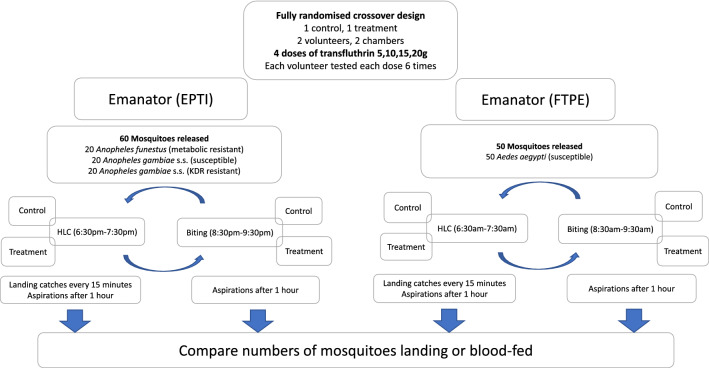
Flow chart showing the various iterations of the experiments conducted in this study

**Fig. 3:**
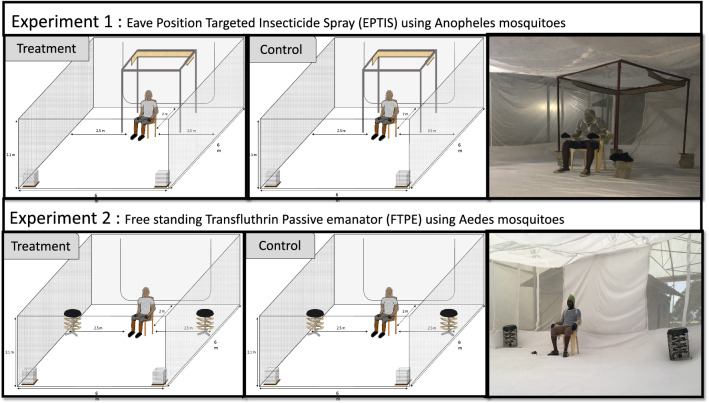
Schematic representation of the I-LACT used for the experiments. **a** Setup of the experiment with transfluthrin-impregnated eave-positioned targeted insecticide (*EPTI*) strips against* Anopheles* mosquitoes. **b** Setup of the experiment with freestanding transfluthrin passive emanators (*FTPE*) against *Aedes aegypti*

A fully balanced cross-over dose–response experiment was conducted using two I-LACT chambers of the SFS, one for the treatment and one for the control, whereby mosquitoes could interact with the human volunteers (Fig. 2). As previous experiments did not show any difference in the numbers of mosquitoes collected between the chambers, the treated and untreated emanators were fixed to the respective chambers for the duration of the experiment to avoid any potential contamination. Each experimental day, one replicate for biting and one for landing was conducted with the same volunteers. A replicate comprised 1 h of exposure to either the treatment (transfluthrin) or the negative control. To simulate an outdoor peridomestic setting, biting or landing was conducted 2 m from the end inside the I-LACT (Fig. 3). Four doses of transfluthrin-treated emanators (5 g, 10 g, 15 g and 20 g) were evaluated consecutively. Each dose was tested for six replicates, after which the emanator with the next highest concentration of transfluthrin was used.

Two male volunteers aged 25–40 years were recruited by written informed consent. The volunteers were non-smokers and non-alcohol drinkers, and did not use perfumed cosmetics prior to the experiment to minimize heterogeneity in their attraction to mosquitoes [29]. To standardize the area available to the mosquitoes for biting (knees and ankles), the volunteers wore closed shoes and a bug jacket (Fig. 3). The volunteers were rotated between compartments (treatments) after each experimental day (one day for landing and the following day for biting) to account for differential attractiveness to mosquitoes between individuals [30]. Temperature and humidity were recorded inside one of the I-LACT using a Tiny Tag Gemini Data Logger (Chichester, West Sussex, UK). To ensure transfluthrin vaporization, the experiments were conducted at temperatures above 23 ºC [31].

On each experimental day, the treatment and control were allocated to one of the two chambers of the I-LACT 45 min before the experiment commenced, to allow emanation of the transfluthrin to have started before the experiment began. The experiment started when the volunteer sat down on the chair and the mosquitoes were released into the chamber of the I-LACT from the release cages, which were opened by pulling a string (Fig. 3).

#### Outcomes

The primary outcome was recaptured mosquitoes, which was measured as number of HLC with the landing method, and number of blood-fed mosquitoes with the biting method. The secondary outcome was PE, which was measured by comparison of the number of recaptured mosquitoes relative to those of the corresponding control.

### Experiment 1: evaluation of EPTI with different doses of transfluthrin against* Anopheles* mosquitoes

To simulate placement on an eave, the EPTIs were mounted at the top of metal stands measuring 1.6 × 1.6 × 2 m, which were placed inside the cage at 2 m from the volunteer who was seated in front of the cage (Fig. 3). A total of 60 mosquitoes comprising 20 mosquitoes of each of three strains—pyrethroid-resistant *An. gambiae* s.s. (Kisumu strain; KDR), pyrethroid-susceptible *An. gambiae* s.s. (Ifakara strain), and *An. funestus* (FUMOZ strain)—were released per replicate (Fig. 2). On each day of the experiment, one replicate (using the landing or biting method) was conducted between 1830 and 1930 hours, followed by a second replicate, which was conducted between 2030 and 2130 hours. The methods, i.e. landing or biting, were alternated after every three replicates, to ensure that possible differences in host-seeking response of the mosquitoes due to their circadian rhythms could be accounted for.

### Experiment 2: evaluation of FTPE with different doses of transfluthrin against *Ae. aegypti*

Two FTPE were positioned on the ground at 2.5-m distance either side of the volunteer and at 2 m from the back of the chamber (Fig. 3). Fifty pyrethroid-susceptible *Ae. aegypti* mosquitoes (Bagamoyo strain) were then released into the chamber (Fig. 2). A total of three replicates for the biting experiment and three for the landing method were conducted over 3 consecutive days, between 0630 and 0730 hours for the former and between 0830 and 0930 hours for the latter. This order was switched for the remaining 3 experimental days, with the landing method conducted first to control for temporal bias when comparing the results of the two methods, which could have been affected by temperature and mosquito circadian rhythm.

### Biting experiment procedure

On each day of the experiments, one volunteer was assigned to either the treatment or the control chamber. During the experiments, the volunteer sat on a chair and the mosquitoes were allowed to fly freely and feed in the area between the knee and the ankle [32]. At the end of the period of exposure, the mosquitoes were collected from within the netting chamber for 45–60 min. All knocked-down and resting mosquitoes were located (head torches were used for this at night) and aspirated from the floor and walls of the I-LACT chamber, using mouth aspirators, and then placed in paper cups, with no more than 25 mosquitoes per cup to minimize the mortality that can occur when mosquitoes interact with one another at high densities. The mosquitoes were immediately transported to the insectary and scored as fed or unfed.

### Landing experiment procedure

On each day of the experiments, one volunteer was assigned to either the treatment or the control chamber. Volunteers assigned to the control were not allowed to enter the treated compartment for any reason before the experiment commenced. The volunteers gently aspirated the mosquitoes that landed on them in the area between the knee and ankle by using mouth aspirators (HLC method). These mosquitoes were placed into a paper cup; a fresh cup was used after every 15-min collection period. After each 15-min collection period, the paper cups were placed in a sealed plastic container to avoid exposure of the mosquitoes to transfluthrin; thus, the mosquitoes were effectively removed from the experiment upon collection. The experiment ended after 1 h, and any remaining mosquitoes were collected by aspiration and placed into cups. All the cups containing mosquitoes were transported to the insectary for counting and recording other data. Head torches were worn to locate and collect *Anopheles* mosquitoes when experiments were conducted in the evening.

### Data analysis

#### WHO susceptibility bioassays using transfluthrin

Data from the WHO susceptibility tests are reported as the mean percentage 24-h mortality of the four replicates. Probit regression analysis was used to calculate the DC for transfluthrin from the lethal dose (LD) required to kill 99% of mosquitoes (LD99), where the DC is equivalent to 2 × LD99.

#### Comparison of the number of recaptured mosquitoes between the biting and landing methods

Analyses of the experimental data were done in Stata 14 (Stata Corp) statistical software [33]. Descriptive analyses were conducted to generate the mean proportion of fed or landed mosquitoes with the respective 95% confidence interval (CI), which are presented in the graphs.

To compare biting and landing in the treatment and control, the number of mosquitoes caught using HLC in the landing experiment and the number of mosquitoes that fed in the biting experiment were merged to create a single variable named ‘recaptured’. Recaptured mosquitoes were modelled using negative binomial probability distributions with the logit link function. The collection method (landing vs biting), treatment, dose, volunteer, and mosquito species were treated as independent categorical fixed effects. Temperature and humidity were added to the model as continuous variables. The PE were calculated from the relative risk (RR), using the formula (1 − RR).

Also, the comparison of biting and landing mosquitoes at different doses was assessed using negative binomial probability distributions with the logit link function. The number of fed or landed mosquitoes, treatment, dose, volunteer, and mosquito species were treated as independent categorical fixed effects. Temperature and humidity were added to the model as continuous variables. The PE were calculated from the RR using the formula (1 − RR).

Additionally, for comparison between the biting and landing methods, Bland–Altman plots were used to assess the agreement of the PE measured by the two collection methods and to examine any systematic difference (fixed bias) between the measurements [26].

## Results

### WHO susceptibility bioassays using transfluthrin

A clear dose–response was observed for mortality (Table 2). The final DC for each species was obtained by doubling the estimated LD99 (Table 3). The DC for *An. gambiae* (Ifakara strain) was 0.290%, while for *Ae. aegypti* it was 0.068%. *An. gambiae* (Kisumu strain: KDR) and *An. funestus* (FUMOZ strain) from the laboratory were fully susceptible to transfluthrin at DC 0.29% (> 98% mortality; Table 4).

**Table 2:** Knock-down and 24-h mortality responses of laboratory strains of *Anopheles gambiae* sensu stricto (s.s.) (Ifakara strain) and *Aedes aegypti* (Bagamoyo strain) exposed to various concentrations of transfluthrin-treated paper

Mosquito species	Transfluthrin concentration (%)	No. of mosquitoes exposed	Knock-down (%)	24-h mortality (%)
*An. gambiae*	0.00125	100	0	2
	0.0025	100	3	2
	0.005	100	7	3
	0.01	100	81	63
	0.02	100	77	49
	0.04	100	100	69
	0.08	100	100	87
	0.1	100	100	100
*Ae. aegypti*	0.003125	100	0	0
	0.00625	100	9	5
	0.0125	100	38	40
	0.025	100	96	96
	0.05	100	100	100

**Table 3:** Discriminating concentration (*DC*) of transfluthrin-treated paper for *Anopheles gambiae (*Ifakara strain) and *Aedes aegypti* (Bagamoyo strain)

Mosquito species	LD50	LD99	DC
*An. gambiae* s.s.	0.0145 (0.013–0.016)	0.145 (0.103–0.187)	0.290
*Ae. aegypti*	0.0132 (0.012–0.0142)	0.034 (0.028–0.040)	0.068

*LD* Lethal dose,* LD50* dose required to kill 50% of the mosquitoes,* LD99* dose required to kill 99% of the mosquitoes

**Table 4:** Susceptibility of *Anopheles gambiae* (Kisumu strain; KDR) and *Anopheles funestus* (FUMOZ strain) exposed to experimentally established DCs of transfluthrin

Mosquito species	DC	No. exposed	Knock-down at 60 min (%)	24-h mortality (%)
*An. gambiae* s.s.	0.290	100	100	99
*An. funestus*	0.290	100	100	100

### Environmental conditions

During the experiments with *Anopheles* mosquitoes, the average temperature was 25.5 °C (24.5–27 °C) and the average RH 70.2% (61.7–76.1%). For the experiments with *Aedes* mosquitoes, the average temperature was 27.1 °C (25.7–28.5 °C) and the average RH 90.0% (89.0–90.8%). We were unable to measure the airflow inside the I-LACT chamber with the anemometer located at the site.

### Recapture of mosquitoes in the I-LACT

For all the experiments and all the mosquito strains, the rate of recapture in the I-LACT was higher than that usually observed for the entire compartment of the SFS. For *Anopheles* mosquitoes, recapture was 427/480 (89%) in the treatment and 453/480 (95%) in the control. For *Ae. aegypti*, recapture was 1445/1600 (90%) in the treatment and 1565/1600 (98%) in the control.

### Comparison of number of recaptured mosquitoes between collection methods

In the presence of transfluthrin, fewer female *Anopheles* mosquitoes (Ifakara, Kisumu and FUMOZ strains) were caught using the biting compared to the landing method [incidence rate ratio (IRR) = 0.82, 95% CI 0.74–0.91, *P* < 0.0001]. A similar, but less pronounced, difference was seen between the biting and the landing methods for the controls (IRR = 0.90, 95% CI 0.82–0.97, *P* < 0.001) (Table 5).

**Table 5:** Summary of the results for the evaluation of different doses of transfluthrin used in emanators across different species of *Anopheles* mosquitoes in the Ifakara large ambient chamber test, as measured using a landing method (human landing catch; HLC) and a biting method (blood-feeding)

Mosquito species	Dosage	Landing experiment^a^			Biting experiment^b^			IRR landing vs biting	
		IRR (95% CI)	*P*-value	%PE (1-1RR)	IRR (95% CI)	*P*-value	%PE (1-1RR)	IRR (95% CI)	*P*-value
Overall	Overall							0.87 (0.81–0.93)	< 0.001
	Control							0.90 (0.82–0.97)	0.01
	Transfluthrin							0.82 (0.74–0.91)	< 0.001
*Anopheles gambiae* (Ifakara strain)	0 g	1.00			1.00			1	
	5 g	0.65 (0.47–0.88)	0.01	35	0.60 (0.44–0.82)	0.01	40	0.77 (0.63–0.94)	0.01
	10 g	0.55 (0.40–0.72)	0.01	45	0.46 (0.33–0.66)	0.01	34		
	15 g	0.52 (0.38–0.72)	0.01	48	0.51 (0.36–0.74)	0.01	49		
	20 g	0.41 (0.28–0.61)	0.01	59	0.31 (0.11–0.51)	0.01	69		
*Anopheles gambiae* (Kisumu strain; KDR)	0	1.00			1.00			1	
	5 g	0.56 (0.41–0.77)	0.01	44	0.68 (0.50–0.93)	0.01	32	0.97 (0.80–1.17)	> 0.05
	10 g	0.59 (0.43–0.81)	0.01	41	0.57 (0.41–0.81)	0.01	43		
	15 g	0.46 (0.32–0.65)	0.01	54	0.48 (0.34–0.69)	0.01	52		
	20 g	0.44 (0.32–0.64)	0.01	56	0.46 (0.31–0.69)	0.01	54		
*Anopheles funestus*	0	1.00			1.00			1	
	5 g	0.76 (0.57–1.00)	0.01	34	0.70 (0.53–0.95)	0.01	30	0.75 (0.63–0.89)	< 0.001
	10 g	0.70 (0.52–0.92)	0.01	30	0.56 (0.40–0.77)	0.01	44		
	15 g	0.68 (0.50–0.90)	0.01	32	0.50 (0.36–0.69)	0.01	50		
	20 g	0.43 (0.30–0.62)	0.01	57	0.40 (0.26–0.60)	0.01	60		

Incidence rate ratio (*IRR*) was adjusted for temperature, humidity, volunteer, and compartment

*CI* Confidence interval

^a^Estimated by comparison between the treatment and the control for each dose of transfluthrin used in the HLC method

^b^Estimated using the model comparing the transfluthrin treatment and control for the biting method, in which the mosquitoes were allowed to interact with the human volunteers and blood-feed

**Fig. 4:**
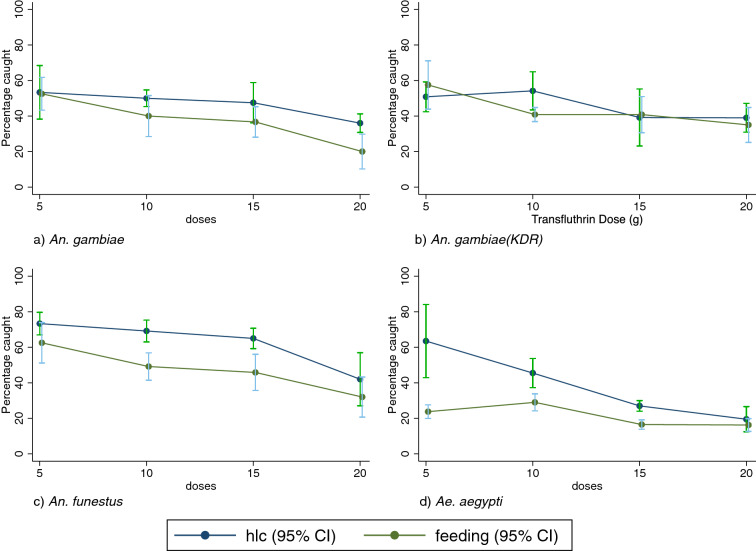
Proportion of recaptured mosquitoes using the HLC method or the biting method for all species and strains of mosquitoes used in this study

With respect to species effects (Fig. 4; Table 5), the overall proportion of mosquitoes caught when they were feeding was lower than that of mosquitoes recaptured by HLC for *An. gambiae* s.s. (IRR = 0.77, 95% CI 0.63–0.94, *P* < 0.01) and *An. funestus* (IRR = 0.75, 95% CI 0.63–0.89, *P* < 0.001). The data were not significantly different for *An. gambiae* s.s. (Kisumu strain) (IRR = 0.97, 95% CI 0.80–1.17, *P* > 0.05).

For *Ae. aegypti* mosquitoes, there was a greater overall difference in the proportion of recaptured mosquitoes between the biting and landing experiments (IRR = 0.63, 95% CI 0.57–0.70, *P* = 0.001). The results of the landing and biting experiments were significantly different both for the treatment (IRR = 0.56, 95% CI 0.46–0.67, *P* = 0.01) and the control (IRR = 0.70, 95% CI 0.64–0.76, *P* = 0.001) (Table 6).

**Table 6:** Summary of the results for the evaluation of different doses of transfluthrin used in emanators for* Aedes aegypti* mosquitoes in the I-LACT as measured using a landing method (HLC) or a biting method (blood-feeding)

Mosquito species	Dosage	Landing experiment^a^			Biting experiment^b^			IRR landing vs biting	
		IRR (95% CI)	*P*-value	%PE (1-1RR)	IRR (95% CI)	*P*-value	%PE (1-1RR)	IRR (95% CI)	*P*-value
*Aedes aegypti*	Overall							0.87 (0.81–0.93)	0.01
	Control							0.90 (0.82–0.97)	0.01
	Transfluthrin							0.82 (0.74–0.91)	0.01
*Aedes aegypti*	0 g	1.00			1.00			1.00	
	5 g	0.74 (0.63–0.87)	0.01	26	0.47 (0.32–0.60)	0.01	53	0.63 (0.57–0.70)	0.01
	10 g	0.66 (0.52–0.85)	0.01	44	0.38( 0.31–0.47)	0.01	62		
	15 g	0.37 (0.28–0.49)	0.01	67	0.24 (0.18–0.31)	0.01	74		
	20 g	0.27 (0.19–0.36)	0.01	73	0.25 (0.19–0.32)	0.01	75		

IRR adjusted for temperature, humidity, volunteer, and compartment

^a^Estimated by comparison between the treatment and the control for each dose of transfluthrin used in the HLC method

^b^Estimated using the model comparing the transfluthrin treatment and control for the biting method, in which the mosquitoes were allowed to interact with the human volunteers and blood-feed

**Fig. 5:**
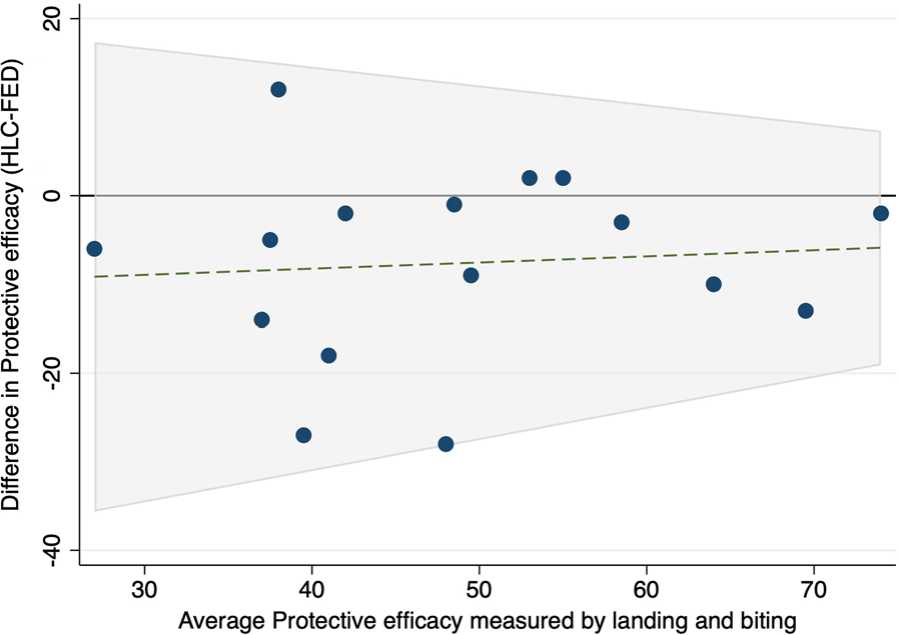
Bland–Altman comparison of protective efficacy determined through landing or biting methods

The Bland–Altman plot (Fig. 5) of the PEs showed that there was consistent agreement in the results between the biting and landing methods. For *Anopheles* mosquitoes, the mean difference was −4.75, and the limits of agreement were between -25.57 and 16.07. While the overall difference in PE measured by landing was similar to that of biting, and there was no systematic bias between the methods, the limits of agreement were wide, indicating that precise estimates of feeding inhibition are not possible with the HLC method. The difference was reduced as the average measured PE of the intervention increased, indicating that the results of the two methods were more similar when the interventions were more efficacious (Fig. 5).

### PEs of different doses of transfluthrin against *Anopheles and Aedes* mosquitoes measured by the landing and biting method

Overall, a clear dose response in PE was observed for all of the species for both of the methods. A higher PE was determined using the biting method compared to the landing method for *An. gambiae* s.s., *An. funestus* and *Ae. aegypti*; this difference was particularly pronounced for *Ae. aegypti* at low concentrations of transfluthrin. However, the relative difference decreased at higher transfluthrin concentrations, and when transfluthrin was applied at a dose of 20 g, there was no difference in the calculated PE between the methods for any of the species (Fig. 4).

## Discussion

### Comparison between landing and biting methods for the measurement of the PE of transfluthrin

The HLC method is the gold standard for the measurement of human exposure to vectors, and has been extensively used for the evaluation of different vector control tools [17]. The human landing rate gives an approximation of the number of mosquitoes that could bite one person at a particular time and place [34, 35]. For vector-borne pathogens, vector bites are critical for disease transmission, and these and daily mosquito mortality are the most important parameters for the determination of disease risk through mathematical modelling [36].

There was evidence that transfluthrin induced feeding inhibition, as the difference in biting compared to landing was greater in the transfluthrin arm than in the control arm at the lower doses. However, the PE measured by the landing method and the biting method broadly agreed across all the species and doses tested. Differences between the results of the methods were smallest at the highest (most effective) transfluthrin doses for *Ae. aegypti* and *An. gambiae* (Kisumu strain; KDR). While there were differences in the results between the landing and biting experiments, the Bland–Altman plots showed that there was good agreement between the PE measured by each method. We therefore suggest that HLCs are a reasonable proxy for bites, and can be used as a substitute for blood-feeding in field evaluation of transfluthrin to limit the risk of vector-borne disease transmission [37].

A higher proportion of* Anopheles* mosquitoes were caught with the landing method than with the biting method. This was also consistently the case for *Ae. aegypti* when the methods were compared across doses of transfluthrin. However, this difference was not as pronounced with *An. gambiae* Kisumu strain (KDR), which is a pyrethroid-resistant mosquito. However, this resistance does not greatly impact landing behaviour in the setting used here [27], thus, this lack of difference could have been due to chance. Further evaluation of landing versus biting methods are ongoing, using formulated products in semi-field and experimental hut experiments, to see if they confirm the findings of this study. The differences between biting and landing observed for other mosquito vectors in the presence of transfluthrin may be explained by behavioural modifications, in that mosquitoes may land but are inhibited from feeding due to sublethal effects on odour processing. Several studies have reported feeding inhibition induced by volatile pyrethroids [38, 39] and pyrethrum [40], and it has been hypothesized that the former interact with olfactory sensors and thus alter a mosquito’s ability to feed [41]. Laboratory studies employing membrane feeding have also shown significant reductions in host-seeking behaviours (landing, probing, and blood-feeding) of *Ae. aegypti* exposed to transfluthrin passive emanators [42]. A recent study on caged *Ae. aegypti*, using metofluthrin passive emanators, showed a reduction in mosquito probing rates, used as a proxy for biting, which was dose-dependent [43].

### Use of the I-LACT bioassay for the measurement of additional endpoints

The SFS provides a simulated user environment where the initial evaluation of both outdoors and indoor bite prevention interventions can be performed [44]. However, previous studies have shown that, when the whole compartment of the SFS is used, recapture of the released mosquitoes is below 100% [14, 45–47]. When some of the exposed mosquitoes are not recovered, they are therefore not accounted for in the statistical analysis, which may bias the results. The I-LACT was designed for the evaluation of outdoor vector control tools, particularly those with multiple actions beyond reducing mosquito landings, such as feeding inhibition, knock-down, and delayed mortality, in an attempt to address this problem.

The I-LACT has sides composed of netting that serves to equalize the climatic conditions between the inside and the outside of the chamber. Its ground area, 30 m^2^, represents that of a typical peridomestic space [23], the area within which the tested intervention would be deployed. Furthermore, the I-LACT is large enough to accommodate human volunteers, to allow human-mosquito interaction. This interaction is important as it mimics what happens during host searching, unlike the arm-in-cage experiment in which mosquitoes are placed close to an individual’s arm [48], or where mosquitoes are confined to small cages [23] for the assessment of delayed mortality caused by insecticide exposure, which may bias results. For example, when mosquitoes are held in a space close to the emanator, their mortality will increase [49], and will likely be higher than that when they are free to fly away from the source of the insecticide. The I-LACT may also be a useful bioassay for the evaluation of other outdoor vector control tools which lead to multiple responses, including knock-down, mortality, and blood-feeding inhibition during host seeking. It also allows the use of consistently high numbers of disease-free mosquitoes in semi-field experiments to ensure that the statistical power is high.

Around 90% recapture of released mosquitoes was demonstrated with the I-LACT bioassay. This high recapture rate provides an opportunity to fully assess the multiple effects of volatile pyrethroids on exposed mosquitoes. Volatile pyrethroids exert several measurable outcomes on exposed mosquitoes, including repellence [50], blood-feeding inhibition [42], disarming [16], knock-down (sublethal incapacitation) [39], and mortality [39, 51]. Of these outcomes, only repellence can be appropriately evaluated by HLC, as only mosquitoes that land are taken into consideration in the analysis. Other outcomes such as mortality or knock-down may not be fully assessed by HLC [28, 45], as mosquitoes will spend more time in contact with the treated device while blood-feeding, which may increase mortality. Conversely, blood-fed mosquitoes show enhanced survival when exposed to pyrethroids [52]. While these additional endpoints are routinely assessed in experimental hut trials of pyrethroids that are applied to insecticide-treated nets [53] and correlate with the results of clinical trials, guidelines for ambient emanators and mosquito coils [54], as well as spatial repellents [55], mainly focus on mosquito landing. Measuring these additional endpoints is important for understanding the full impact of VPSRs when applied at scale, and may be used for mathematical modelling [56] to better understand target product profiles and entomological correlates of impact.

The importance of multiple endpoints of transfluthrin treatment beyond bite prevention alone was demonstrated in a randomised control trial (RCT) in Indonesia, where there was no significant protection from mosquito landings offered by transfluthrin emanators compared to the control, yet clinical cases of malaria were significantly reduced [57]. These findings suggest that there are some limitations to using only HLC to measure the efficacy of volatile pyrethroids in the field, and further endpoints should be evaluated in RCTs of volatile pyrethroids, including human blood index [58] as a proxy for blood-feeding inhibition, and population survival estimates as a proxy for mortality [59]. A recent cluster-randomised trial of a passive transfluthrin emanator in Iquitos, Peru demonstrated a reduction in arbovirus incidence as well as in *Ae. aegypti* abundance and proportion of blood-fed mosquitoes [60], suggesting the importance of mortality and blood-feeding inhibition for public health applications of volatile pyrethroids.

### Estimates of the PEs at different doses of transfluthrin measured by the landing or biting method

The I-LACT was used to carry out a dose–response experiment designed to compare the PEs of different doses of transfluthrin, as determined by using a landing or biting method. A short exposure time was used in the experiments to mimic real life, as mosquitoes are likely to be exposed to a treatment for only a short period of time before their behavioural responses are elicited [41]. There was no interaction between treatment and species, indicating that transfluthrin used at the concentrations in this experiment induced protection against all the mosquito species tested, regardless of their resistance mechanism, in agreement with previous work [27]. The calculated PE was similar between the landing and biting experiments. The findings from this study agree with those from a field study undertaken in Tanzania by Ogoma et al*.* [31], who showed that hessian strips treated with transfluthrin at doses of between 5 and 15 g reduced the number of mosquito landings in the peridomestic space similarly for several *Anopheles* vector species. These results indicate that, in an area where mosquitoes bite outdoors, fabric treated with the lower dose could be used to both protect humans from mosquito bites and provide community protection, while maximizing human safety. A consistent PE of 30% over a period of several months achieved with a product with a high compliance of use would confer greater protection than use of a product with a higher PE but a low compliance of use [61].

### Effect of volume on the evaluation of the volatile pyrethroid

The PE of around 30% against *Ae. aegypti* and *Anopheles* achieved with transfluthrin at the lowest dose of 5 g in the present study was lower than the 60% estimated using hessian strips at the same dosage in a previous experiment, which was conducted using the landing method in the entire SFS compartment [27]. A PE of 60% was replicated in a field and semi-field experiment conducted in Kenya [47]. The difference in the PEs may be explained by the difference in volume between the I-LACT and the semi-field compartment. The volume of the I-LACT into which the mosquitoes were released was 75.6 m^3^, whereas the larger 1228-m^3^ volume of each semi-field compartment allowed the mosquitoes to move further away from the source of transfluthrin. Similarly, a study conducted to measure the PE of a topical repellent in the SFS (here considered to be a relatively small volume) and in the field (here considered to be a relatively large volume) reported a higher PE in the field trial [15]. These results indicate that it is likely that the chance of repeated biting by a mosquito in an area with a large volume are reduced because the mosquito may move away from the host after coming into contact with transfluthrin. This also suggests that, in a smaller space, inhibition of landing could be underestimated, and sublethal incapacitation and mortality could be overestimated, as the modes of action are dose dependent, with mortality occurring at higher doses or longer exposure time [22].

### Effect of climatic conditions on the efficacy of the volatile pyrethroid

The PE was slightly higher for both the landing and biting methods for *Aedes* mosquitoes compared to *Anopheles* mosquitoes. These differences in protection may have been partly due to the differences in ambient temperature at the time that the two experiments were conducted. The ambient temperature was slightly lower (25 °C) in the night-time experiment with* Anopheles* mosquitoes than during the experiment conducted in the morning with *Aedes* mosquitoes (27 °C). However, these temperatures fall within the range, i.e. 21–30 °C, at which the effect of transfluthrin is optimal [31]. Future experiments should be designed to evaluate the efficacy of transfluthrin-treated emanators at different temperatures, and environmental conditions should always be taken into consideration in the analyses. Although the wind speed inside the SFS could not be measured in the present study because it was below the limit of detection of the anemometer used, it is possible that, under conditions of greater air movement and lower temperature, a lower PE would be achieved using the same type of emanator and dosages as used here. In some studies, more consistent evaporation of a volatile pyrethroid between replicates is achieved through the use of a fan [43], and consistency in the rate of evaporation of a tested pyrethroid is an important consideration for future trials of ambient emanators.

## Conclusions

The feeding inhibition of *An. gambiae* s.s., *An. funestus* and *Ae. aegypti* mosquitoes in the presence of transfluthrin was underestimated by the HLC method, and the magnitude of the difference between landing and biting varied among the species and doses of transfluthrin tested in this study. The PE calculated for the landing or biting methods did not show any systematic bias, and was generally in agreement when tested with the Bland–Altman plot, with better agreement at higher concentrations of transfluthrin, which also afforded greater PE. Therefore, either method can be used to assess the personal PE of volatile pyrethroids, with the caveat that results may vary due to the stochasticity inherent to entomological experiments, with greater variability occurring when interventions provide lower efficacy. The findings reported here indicate that HLC can be used as a proxy of personal PE for the evaluation of volatile pyrethroids, especially when the difficulties associated with counting fed mosquitoes in a field setting are taken into account.

## Data Availability

All data generated or analysed during this study are included in this published article and its additional file.

## Abbreviations

CI: Confidence interval
DC: Discriminating concentration
EC: Emulsifiable concentrate
EPTI: Eave-positioned targeted insecticide
FTPE: Freestanding transfluthrin passive emanator
HLC: Human landing catch
I-LACT: Ifakara large ambient chamber test
IRB: Institutional Review Board
PE: Protective efficacy
RH: Relative humidity
SFS: Semi-field system
WHO: World Health Organization
